# GeoAir—A Novel Portable, GPS-Enabled, Low-Cost Air-Pollution Sensor: Design Strategies to Facilitate Citizen Science Research and Geospatial Assessments of Personal Exposure

**DOI:** 10.3390/s21113761

**Published:** 2021-05-28

**Authors:** Yoo Min Park, Sinan Sousan, Dillon Streuber, Kai Zhao

**Affiliations:** 1 Department of Geography, Planning, and Environment, East Carolina University, Greenville, NC 27858, USA; 2 Department of Public Health, Brody School of Medicine, East Carolina University, Greenville, NC 27834, USA; sousans18@ecu.edu; 3 North Carolina Agromedicine Institute, Greenville, NC 27834, USA; 4 Environmental Health Sciences Program, Department of Health Education and Promotion, College of Health and Human Performance, East Carolina University, Greenville, NC 27858, USA; streuberd20@students.ecu.edu; 5 Independent Researcher, Winterville, NC 28590, USA; kai@kaizhao.com

**Keywords:** low-cost sensor, air quality, air sensing, citizen science, personal exposure, wearable devices, geospatial technologies, geographic information systems, particulate matter, volatile organic compounds

## Abstract

The rapid evolution of air sensor technologies has offered enormous opportunities for community-engaged research by enabling citizens to monitor the air quality at any time and location. However, many low-cost portable sensors do not provide sufficient accuracy or are designed only for technically capable individuals by requiring pairing with smartphone applications or other devices to view/store air quality data and collect location data. This paper describes important design considerations for portable devices to ensure effective citizen engagement and reliable data collection for the geospatial analysis of personal exposure. It proposes a new, standalone, portable air monitor, GeoAir, which integrates a particulate matter (PM) sensor, volatile organic compound (VOC) sensor, humidity and temperature sensor, LTE-M and GPS module, Wi-Fi, long-lasting battery, and display screen. The preliminary laboratory test results demonstrate that the PM sensor shows strong performance when compared to a reference instrument. The VOC sensor presents reasonable accuracy, while further assessments with other types of VOC are needed. The field deployment and geo-visualization of the field data illustrate that GeoAir collects fine-grained, georeferenced air pollution data. GeoAir can be used by all citizens regardless of their technical proficiency and is widely applicable in many fields, including environmental justice and health disparity research.

## 1. Introduction

Personal exposure to air pollution occurs through dynamic interactions between an individual and air pollutants [1]. Indoor and outdoor exposure to various air pollutants, such as particulate matter (PM) and volatile organic compounds (VOCs), has detrimental health effects based on the exposure levels and duration [2,3]. Elevated PM exposure is associated with various illnesses, including asthma, stroke, heart attack, and lung cancer, causing increased mortality and morbidity [4]. While the health effects of coarse (PM_10_) and fine particles (PM_2.5_ and PM_1_) differ, smaller particles are known to pose greater health issues as they can travel deep inside the lung tissues. Indoor VOCs can lead to mucous membrane irritation, fatigue, and carcinogenic effects, whereas outdoor ozone formed by VOCs’ reaction with nitrogen oxides and sunlight can increase the risk of asthma development among children [2].

Assessing personal exposure accurately is an important step in taking preventive measures to minimize adverse health outcomes. However, a challenge in personal exposure assessments is that air pollution concentrations are constantly changing over space and time, and individuals (receptors) also move through various geographical areas to live, work, and play [5]. This complex reality of exposure has provoked a paradigm shift from static, residence-based, aggregate-level approaches to the study of environmental exposure/heath to mobility-based, individual-level approaches in recent years [6,7,8]. In this new paradigm, accurate assessments of personal exposure require two critical components: (1) detailed, personal travel-pattern data (e.g., GPS tracking data) and (2) air-quality data collected at a fine spatio–temporal scale [1]. Due to the spatio–temporal aspects of these two components, researchers have increasingly turned to an advanced geographic information system (GIS) and various geographic techniques to integrate the datasets for mapping and geospatial assessments of exposure. Geographic approaches to exposure research can enhance an understanding of where, when, how, and why people experience exposure, allowing researchers to analyze multiple factors simultaneously based on their spatio–temporal relationships.

Recent advancements in air-sensing and positioning technologies have opened new possibilities for collecting both highly localized, real-time air pollution data and individuals’ movement pattern data that allow geospatial assessments of exposure [9,10,11]. In particular, the emergence of low-cost, compact air sensors has made it possible for citizens to participate in community air monitoring or personal air sampling in their places of daily activity [12,13]. In recent years, a growing number of citizen science projects have used low-cost air monitors to provide local air quality data to communities, with the goals of raising public awareness of air pollution, fostering behavioral changes, or empowering communities to take action [14,15,16]. However, several limitations of existing air-sensing devices may hinder effective citizen engagement and successful harvesting of georeferenced air quality data. First, many devices generally do not contain all the components necessary for citizen science projects for geospatial assessments of personal exposure. For example, devices that are designed to mainly target the general public tend to be easy to use and visually pleasing, but they do not provide sufficient accuracy for credible data [16], lack the elements required for scientific research, such as data loggers, and are often suitable for measurement only at a fixed location (e.g., a user’s home). In contrast, devices that are widely used by researchers or scientists are often not ready for citizen science applications due to their poor user-friendliness and technical complexity [17,18]. However, they have been frequently employed in community-based participatory research, despite being designed for researchers, scientists, or those who are technically capable.

Second, many existing devices can only be deployed as stationary devices as they lack a battery and GPS functionality. Therefore, many citizen science projects using low-cost sensors have focused mainly on obtaining outdoor air quality data by requesting volunteers to install sensors in their backyards. The data gathered by the distributed ambient sensor network have often been combined with population data for exposure and health risk assessments. However, given that people spend 93% of their lives indoors on average [19], exposure assessments that focus only on outdoor air quality do not inform individuals of the large portion of their daily exposure and may produce erroneous exposure estimates [20]. Moreover, stationary monitoring often has little impact on people’s awareness of or behaviors to improve their surrounding air conditions because it does not fully satisfy participants’ curiosity [14]. It has been reported that people are more interested in identifying the air quality in their own spaces of daily activity than in the general outdoor areas of their communities [18,21] and thus, are more motivated to change their behaviors to reduce air pollution/exposure when they are informed of the air quality in their immediate surroundings [14].

We argue that using a GPS-enabled portable device is the most effective strategy to increase the public awareness of air pollution and promote changes in behaviors because it can offer citizens a full picture of the air quality that they experience in daily life in various indoor/outdoor environments (e.g., homes, workplaces, grocery stores, and in-vehicle). However, only a few consumer devices or research prototypes are portable because complex design considerations make portable devices difficult to develop. To make them as compact and light as possible, most devices do not have a display screen, a large built-in battery that can last more than 10 h, a GPS module, and a data logger. Rather, they depend on smartphone applications or other mobile devices (e.g., external battery packs) to view and store air pollution data and collect GPS data (extracted from users’ phones). However, such a design often poses usability issues, causing a significant burden on participants and failing to engage citizens effectively, elicit improved environmental literacy, and harvest high-quality data [15,16,17,18,22,23].

To address these limitations, we propose an innovative, GPS-enabled, portable, low-cost air-sensing device that can facilitate citizens’ engagement in personal air monitoring and geospatial assessments of personal exposure. The device proposed in this paper is GeoAir2. Its concept and design build on GeoAir1—a prototype developed by Park [11]—by adding a multi-gas sensor, an LTE modem, and a larger battery, as well as by utilizing a more reliable PM sensor and GPS module. The details of GeoAir1 and its real-world application can be found in Park [11]; a manuscript that presents laboratory experiment results about GeoAir1 (which uses the PMS A003 sensor (Plantower, Beijing, China) is currently under review [24]. From this point forward, GeoAir2 will be called “GeoAir” for convenience. This paper describes the critical design considerations for the development of GeoAir and demonstrates the device’s utility by presenting preliminary laboratory and field data and visualizing the field data on 2-D and 3-D maps. The details of sensor calibration, performance, and complete data analysis are not included in this paper as they will be presented in another manuscript.

## 2. Materials and Methods

### 2.1. Design Requirements

A portable air-monitoring device used for geospatial exposure assessments that involve citizens requires unique design considerations. It should be designed to (1) ensure the successful harvesting of georeferenced air quality data from participants and (2) minimize participants’ burden. To meet the first criterion, the device should integrate the following features: (1) high-quality but low-cost PM- and VOC-sensors developed by an experienced manufacturer of environmental sensors to ensure data accuracy; (2) a built-in data logger to store the complete data and prevent the data loss that can occur when only relying on a smartphone application to store data; (3) GPS and Wi-Fi for outdoor and indoor positioning; and (4) an LTE modem for near real-time, wireless data transmission to ensure continuous data collection.

For the second criterion, the device should be small, light, wearable/portable, and designed both to reduce the complexity/number of tasks required for participants to complete and minimize the number of other devices that need to be carried and manipulated (e.g., smartphones, GPS data loggers, or external battery packs). A key requirement for new technologies adapted to citizens is to maximize ease of use [18]. A common but mistaken assumption is that all participants, regardless of their age, educational attainment, technological literacy, gender, income levels, race/ethnicity, and so forth, are comfortable with new technologies and can easily understand how to manipulate air-sensing devices as researchers or scientists intend [22]. Therefore, instead of requiring users to connect the device to a smartphone application and manipulate it to view their data, having a display screen on the device is ideal because it allows participants to check the air quality easily whenever they are curious about it. It is also preferable if users can charge the device only once at the end of the day and do not need to carry an external battery pack when they are away from home.

To the best of our knowledge, while some products on the market meet the individual aspects of the requirements mentioned above, no existing devices fulfill them all. Many portable air monitors that are commercially available and widely used, such as AirBeam2 (HabitatMap, Brooklyn, NY, USA), Atmotube (Atmotech Inc., San Francisco, CA, USA), or Aeroqual (Aeroqual Limited, Auckland, New Zealand), claim to be an ideal tool for citizen science research. However, numerous community-based projects have faced significant challenges when implementing these devices on a wide scale [15,16,17,18,22,23]. Many existing devices require users to keep the sensor device and their smartphone at a distance of less than 10 m apart to maintain a constant Bluetooth connection between the two devices for viewing or storing data. However, data are often lost when their Bluetooth connection is interrupted. As observed in previous studies, smartphone applications paired with these sensor devices often crash, causing data loss; they also require technical proficiency in users, leading to frustration for research participants and high dropout rates [11,17,18,23]. Furthermore, these mobile applications frequently work only on Android phones and do not function properly on low-quality smartphones, limiting the eligible participants to a subset of the population. Participants must also charge both devices frequently because the GPS running constantly on their phone and the fan in the air sensor cause excessive battery drain. In addition, without a built-in GPS, most devices have to couple with a separate GPS receiver device or users’ smartphones to collect location data that can be linked to air pollution data. These limitations are also present in many research prototypes that were developed by research groups because they are primarily designed for researchers rather than the general public [18]. In the following subsection, we describe how GeoAir can address these limitations and meet all the aforementioned criteria.

### 2.2. Detailed Description of GeoAir Design

The design of GeoAir centers on citizen science research and geospatial assessments of personal exposure. GeoAir integrates the following features: a PM sensor (SPS30, Sensirion, Zurich, Switzerland); a multi-gas, humidity, and temperature sensor combo module (SVM30, Sensirion, Zurich, Switzerland) that contains an SGP30 VOC sensor and an SHTC1 humidity and temperature sensor; an LTE-M and GPS module with an internal antenna (SIM7000A, SIMcom, Shanghai, China); a 4000 mAh lithium polymer battery; an M5Stack microcontroller (Shenzhen, China) powered with an ESP32 system-on-a-chip; and an LCD display that allows citizens to readily check air quality at any time and location. The design includes a belt clip attachment point to allow users to wear the device.

After careful review of existing low-cost PM and VOC sensors, we chose the SPS30 PM sensor and SGP30 VOC sensor as candidates for our GeoAir design due to their high accuracy, low cross-unit variability, and long-term stability, as reported in the literature [25,26,27] and in the field evaluation conducted by the Air Quality Sensor Performance Evaluation Center (AQ-SPEC) in the South Coast Air Quality Management District (SCAQMD, USA) [28]. The ease of software integration between SGP30 and SPS30 was another reason that both sensors were selected from the same manufacturer. As the SPS30 is a relatively new optical particle counter that was released on the market in late 2018, there are only a few studies that evaluated the sensor’s performance. However, the laboratory experiments performed by the authors also indicate that the SPS30 sensor outperforms other low-cost sensors in terms of accuracy, biases, and precision for different aerosol types, including salt. A manuscript that presents the results of these laboratory experiments is currently under review [24].

The SPS30 sensor uses the principle of laser-scattering and provides real-time PM mass concentrations for different sized fractions (PM_1_, PM_2.5_, PM_4_, and PM_10_). The sensor has a lifetime of more than 10 years with continuous use and requires no maintenance due to its unique contamination-resistant technology [29]. The SGP30 measures total VOCs (tVOCs), which indicates the total concentration of multiple organic chemicals found simultaneously, mostly as gases in the air. The SGP30 greatly improves upon existing consumer VOC sensors by addressing multiple limitations. It has significantly enhanced long-term stability and sensor accuracy because it uses a siloxane-resistant technology that prevents the deterioration of the sensor accuracy over time due to contamination by siloxanes [26]. In addition, it is suited for mobile applications due to its size and low cost. More details about the hardware interfaces and electrical specifications of SPS30 and SGP30 are available in the datasheets of SPS30 [29] and SVM30 (a module that integrates SGP30), respectively [30].

**Figure 1:**
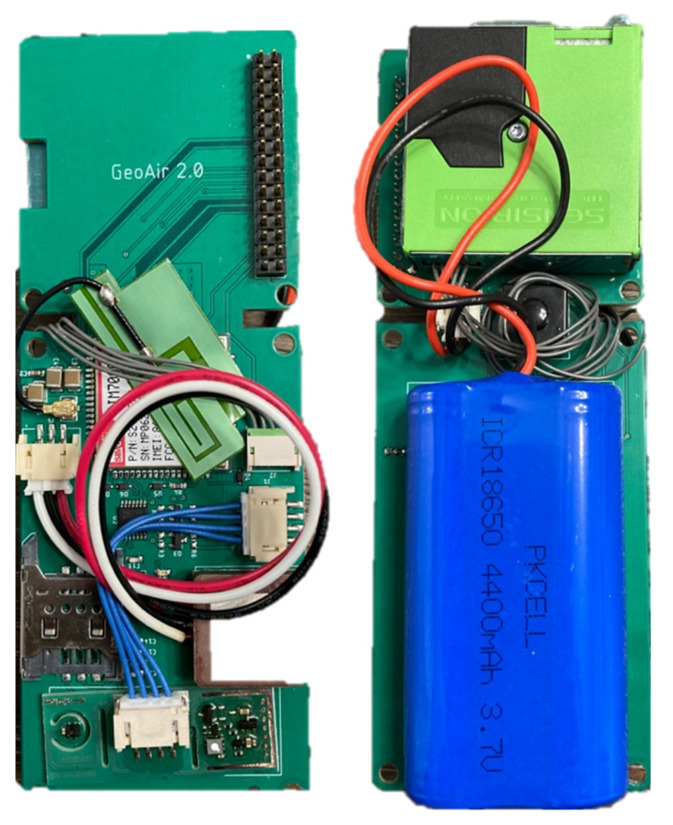
A printed circuit board (front and back) of GeoAir.

**Figure 2:**
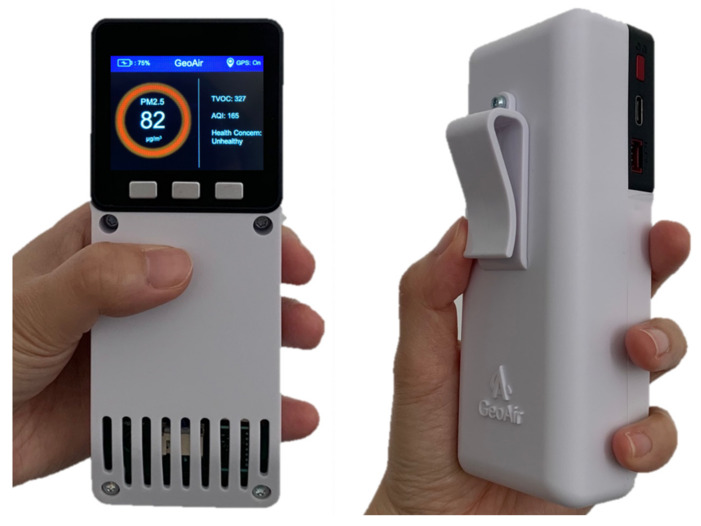
GeoAir2: A portable, GPS-enabled, low-cost air pollution monitor.

A printed circuit board (PCB) was designed and manufactured to integrate all the components into a single package (Figure 1). This package was encased in an injection-molded plastic box (Figure 2). The process of prototyping and manufacturing was completed by Jaycon Systems (Florida, United States) and the cost per unit is USD 250~350. It should be noted that the unit cost is dependent on the volume of units produced.

GeoAir records the following data every minute by default: (1) mass concentrations (μg/m^3^) of PM_1_, PM_2.5_, PM_4_, and PM_10_; (2) number concentrations of PM_0.5_, PM_1_, PM_2.5_, PM_4_, and PM_10_; (3) tVOCs (ppb); (4) temperature (°F); (5) relative humidity (0–100%); (6) geographic coordinates of GPS locations (latitudes and longitudes); (7) timestamps (in Greenwich Mean Time); battery levels (%); (8) a list of Wi-Fi media access control (MAC) addresses for geolocation; (9) and the number of GPS satellites currently with position fixes. Although the SGP30 sensor produces the output for H_2_-based CO_2_eq, GeoAir does not record this output due to insufficient accuracy because it is an estimate based on a hydrogen measurement rather than a real CO_2_ measurement. The manufacturer also does not recommend using the SGP sensors for applications in which real CO_2_ detection is required. In addition, although the default time interval was one minute, the device can be set differently according to study purposes. In this paper, we used a one second interval to obtain finer-scale data because a long battery life was not needed for our preliminary laboratory and field tests. On the other hand, in a study that aimed to estimate “daily” exposure and requires participants to carry the device for several days, a one-minute recording interval may be more appropriate because long battery life is important to reduce the burden for participants.

To prevent data loss and protect data confidentiality and geoprivacy, GeoAir writes data to an encrypted CSV file that is stored on a built-in micro Secure Digital (SD) card and uploads data every hour to a preset URL using HTTPS. The LTE modem is also utilized for real-time telemetry to ensure that the device is being used as intended (e.g., whether the device is turned on and charged). For example, the LTE modem sends a POST request to a URL when the battery life is below 25%. At this time, the device displays a notification to alert users. One of the most important design considerations of GeoAir is to maximize the battery life. The device was designed to last up to 15 h when fully charged by reducing the frequency of data polling to one minute, turning off the PM sensor’s fan when it is not measuring particles between minutes, minimizing the brightness of the display when the screen is not in use, and turning off the LTE modem except when uploading the previous hour of data in bulk to a preset URL once every hour. When the PM sensor wakes up again for the next-minute measurement, the device warms up the sensor for at least 20 s before the measurements to obtain stable outputs, as recommended by Sensirion.

### 2.3. Sensor Performance Evaluation Using Reference Instruments

**Figure 3:**
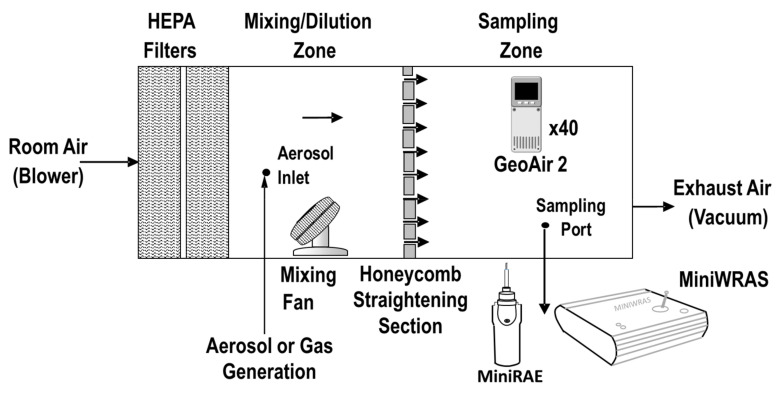
Experimental setup used to test the GeoAir functionality and response for aerosol and VOC.

We tested 40 units of GeoAir for aerosol and gas functionality and response inside a laboratory exposure chamber (Figure 3). The chamber was split into two equal sections: a mixing zone, where aerosol was generated, and a sampling zone, where the sensors were tested. The chamber dimensions are 1.2 m × 0.64 m × 0.64 m (length, width, and height, respectively), and the mixing and sampling zones were separated with a honeycomb straightening section (AS100, Rusken, Kansas City, MO, USA). The 40 GeoAir units were positioned inside the sampling zone. A Mini Wide Range Aerosol Spectrometer (MiniWRAS 1371, cost = USD 30,000, GRIMM Aerosol Technixk GmbH & Co. KG, Ainring, Germany) was used as an aerosol reference instrument. The MiniWRAS can capture the whole range of particle size distribution. It measures 41 bin sizes between 10 nm and 35 µm and can derive PM_1_, PM_2.5_, and PM_10_ measurements in real time. The MiniWRAS combines electrical and optical particle detection, while the SPS30 only uses optical detection. As a VOC reference instrument, a MiniRAE 3000+ PID (Honeywell, Charlotte, NC, USA) was used. The MiniWRAS and MiniRAE were positioned outside the chamber and measured air directly from the sampling zone. Particle free air was supplied to the mixing zone using a 1/4 hp blower (Dayton 7AT80, Grainger Global Sourcing-Motors, Lake Forest, IL, USA) that passes through two high-efficiency particulate absorbing (HEPA) filters (Model: 2GHH1, 99.99% Filter Efficiency, Flanders Corporation, Washington, NC, USA) in series. The exposures (aerosol or gas) were generated in the mixing zone, where two fans were used to mix the air. A vacuum (DM 3000P, Fantech, Lenexa, KS, USA) was used after the sampling zone to ensure the proper disposal of the aerosol and gases generated in the chamber. The GeoAir devices and the MiniRAE were set to record every one second, and the MiniWRAS was set to record every one minute. Two experiments were conducted—the first by generating salt aerosol to test the SPS30 sensors and the second by generating VOC to test the SGP30 sensors.

#### 2.3.1. Aerosol Generation

Salt particles were generated using a 2% by weight prepared salt solution (Sodium Chloride, S9625-1KG, Sigma-Aldrich, St. Louis, MO, USA) and a vibrating mesh nebulizer (Aeroneb Solo System, Aerogen, Galway, Ireland). Different mass concentrations were achieved by operating the nebulizer with a voltage regulator. The salt particles generated by the nebulizer were dried by passing the air stream through a silica bed (Droplet Measurement Technologies, Longmont, CO, USA). The salt particles were then diluted in the mixing zone before passing through the honeycomb straightening section. The mass concentration changed gradually from 0 to 250 µg/m^3^ over 90 min, according to the MiniWRAS, which was monitored in real time. Particle-free mass concentration was established before the experiment and tested using the MiniWRAS.

#### 2.3.2. Gas Generation

Butanol solution (1-BUTANOL, HPLC GRADE, A383, ThermoFisher Scientific, Fair Lawn, NJ, USA) was used to generate VOC inside the chamber. The MiniRAE was calibrated with a 100 ppm Isobutylene calibration gas (SDS 3025, Safeware, Inc., Lanham, MD, USA) before the experiment and set to 1-Butanol. The Butanol vapor was generated using a syringe pump (Aladdin-1000NS, World Precision Instruments, Sarasota, FL, USA) and a 60 mL syringe attached directly to the mixing zone of the chamber. The syringe pump was set at a flow of 10 mL/min for 5 min, then lowered to 0.2 mL/min.

#### 2.3.3. Data Analysis

The GeoAir data were averaged over one minute and time-paired with the MiniWRAS and MiniRAE using MATLAB R2020b. The GeoAir data for the 40 sensors and the MiniRAE data were averaged for each minute, and the standard deviation was calculated. Scatter plots between the MiniWRAS and the average GeoAir data were derived for PM_1_, PM_2.5_, and PM_10_. A scatter plot between the MiniRAE and the averaged GeoAir data for the VOC measurements was also created. The slopes, intercepts, and coefficients of determination (R^2^) for each scatter plot were derived.

### 2.4. Field Deployment and Geovisualizations Using GIS

A volunteer conducted field data collection in the city of Greenville (NC, USA) on 10 March 2021. Prior to the data collection, the volunteer turned on the device and waited for about one minute until the device established a GPS signal and the sensors warmed up and stabilized. When the device is ready for georeferenced measurements, it displays air quality information on the screen. The volunteer wore three GeoAir units clipped to a belt and walked along the roadways in a high-traffic area in the city for an hour. The authors then downloaded the data from the micro SD cards and calculated the average concentrations of the three monitors for PM_1_, PM_2.5_, and VOC for each second. Using the average values, we created 2-D maps to present spatial distributions of the pollutants along the travel route and 3-D maps to show both spatial and temporal patterns.

## 3. Results and Discussion

### 3.1. PM Sensor Performance

**Figure 4:**
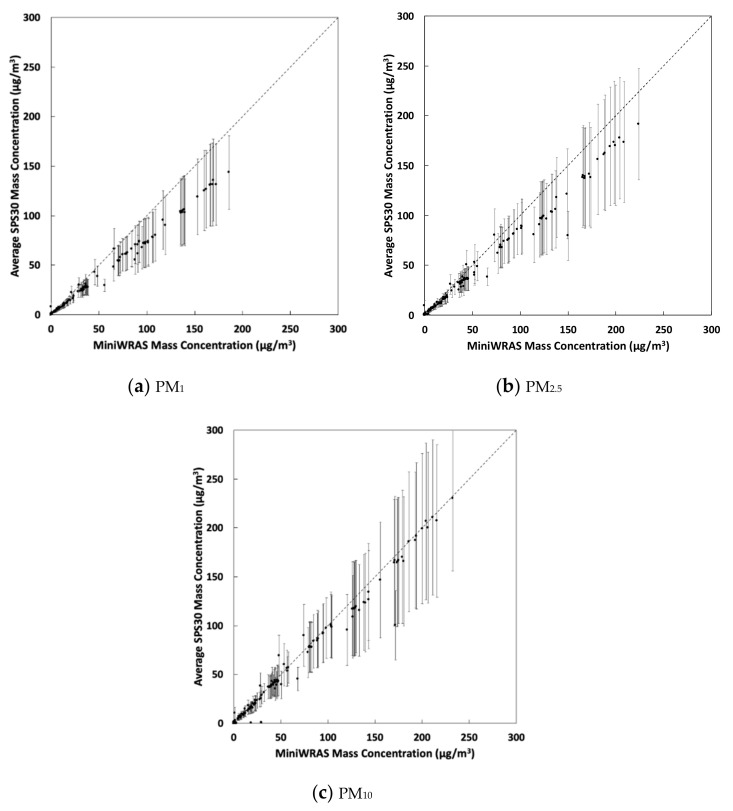
Scatter plots between the average GeoAir and the MiniWRAS data for (**a**) PM_1_; (**b**) PM_2.5_; and (**c**) PM_10_ for varying levels of salt concentration. The *y* axis error bars represent the standard deviation of the GeoAir devices.

Table 1 presents the slopes, intercepts, and R^2^ for PM_1_, PM_2.5_, and PM_10_. Three GeoAir sensors did not provide data and were removed from the analysis. Therefore, the data points represent the average concentration of 37 GeoAir sensors for all particle sizes. The correlation coefficient was high for all particle sizes and ranged from 0.98 and 0.99. The slopes were 0.77, 0.82, and 0.95 for PM_1_, PM_2.5_, and PM_10_, respectively. The intercepts were all below −0.5 μg/m^3^, with the PM_1_ intercept approaching zero. Scatter plots of the average GeoAir data and the MiniWRAS for PM_1_, PM_2.5_, and PM_10_ are shown in Figure 4. The *y* axis error bars represent the standard deviations for all GeoAir sensors. The PM_1_ and PM_2.5_ average mass concentrations for the GeoAir were underestimated compared to the MiniWRAS. PM_10_ average mass concentrations for the GeoAir were close to the one-to-one line, which indicates high precision. However, some of the GeoAir sensors were close to the one-to-one line for PM_1_ and PM_2.5_ concentration, as illustrated by the standard deviation that changed up to ±42, ±62, and ±80 μg/m^3^ for PM_1_, PM_2.5_, and PM_10_, respectively.

Overall, the SPS30 aerosol sensors inside the GeoAir device showed significant correlations with the MiniWRAS reference data for PM_1_, PM_2.5_, and PM_10_. The high correlations may be because the manufacturer calibrates the SPS30 PM sensors with potassium chloride particles [29]. Our result is consistent with a previous study that reported R^2^ = 0.98 for PM_2.5_ when comparing the SPS30 to a high-cost PM reference instrument using ammonium sulfate particles [27].

### 3.2. VOC Sensor Performance

**Figure 5:**
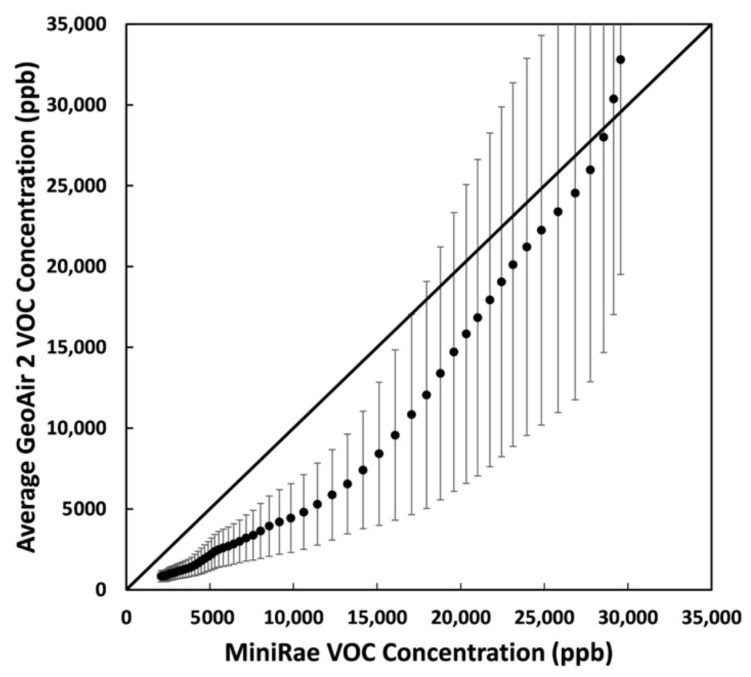
VOC scatter plot for MiniRAE and average GeoAir measurements for 1-Butanol. The *y* axis error bars represent the standard deviation of the GeoAir sensors.

The average concentrations of the GeoAir compared to those of MiniRAE are shown in Figure 5 as a scatter plot. Five GeoAir sensors did not provide data and were removed from the analysis. Therefore, the data points represent the average concentration of 35 GeoAir sensors. The maximum concentrations for the MiniRAE and GeoAir were 29,686 ppb and 46,711 ppb, respectively. The *y* axis error bars represent the standard deviations for all the sensors. The average slope, intercept, and R^2^ values for the GeoAir sensors compared to the reference instrument were 0.99, 3229 ppb, and 0.95, respectively. The GeoAir sensors overestimated VOC levels for concentrations higher than 30,000 ppb and underestimated VOC levels for lower concentrations compared to the estimations of the MiniRAE.

A possible reason that the response time for the MiniRAE is slower than that of GeoAir is that the reference instrument was outside the chamber. The difference in concentrations could be attributed to the calibration. The MiniRAE was corrected for 1-Butanol, whereas the SGP30 sensors were not. However, the calibration factors (slope and intercept) can be used to correct the biases for the GeoAir. According to the manufacturer, the SGP30 sensors were calibrated with ethanol [30]. However, it is unclear why the SGP30 sensors overestimated the VOC concentration for levels above 30,000 ppb. More studies are needed to evaluate the SGP30 sensors against a reference instrument under laboratory conditions.

### 3.3. Preliminary Field Data and Geospatial Mapping

**Figure 6:**
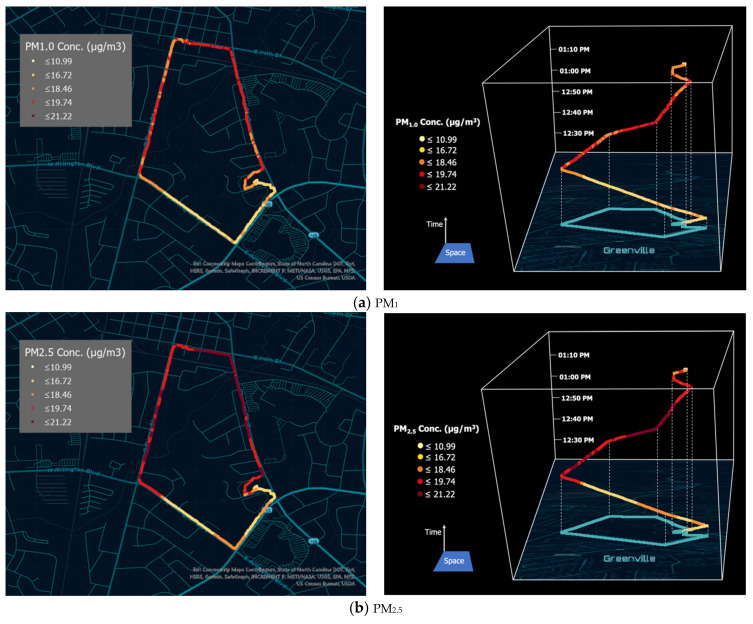

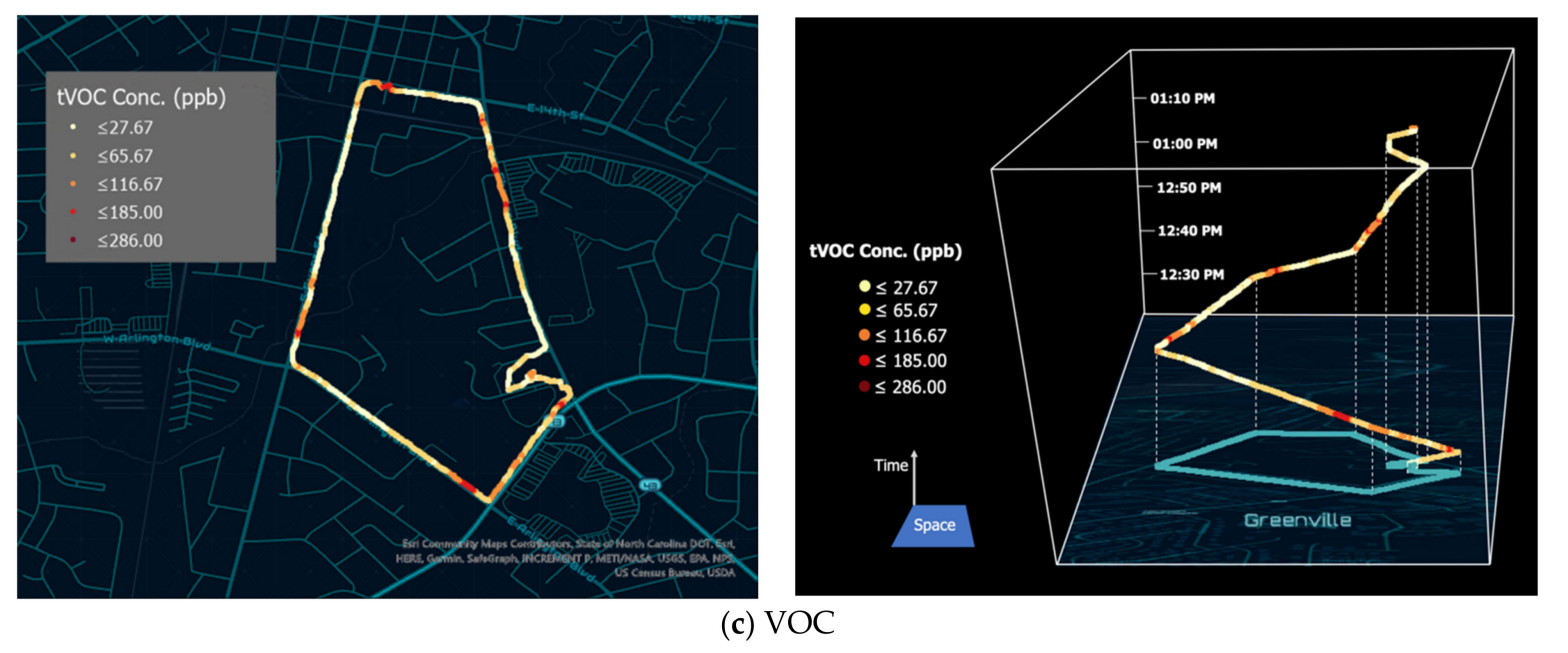
Visualizations of georeferenced air quality data obtained from GeoAir.

The field data contained a total of 2610 measurements for each pollutant (the measurements before the device obtained a GPS signal were excluded). The descriptive statistics of PM_1_ and PM_2.5_ concentrations are as follows, respectively: average, 17.65 μg/m^3^ and 18.66 μg/m^3^; maximum, 20.07 μg/m^3^ and 21.22 μg/m^3^; minimum, 11.09 μg/m^3^ and 11.72 μg/m^3^; and standard deviation, 1.58 μg/m^3^ and 1.67 μg/m^3^. The average PM_2.5_ concentration during the data collection fell into the “moderate” category of the Air Quality Index (AQI), which is the level at which sensitive people, including the elderly, children, and people with respiratory and cardiovascular diseases, may be at risk if they are exposed to this concentration daily over the duration of a year [31]. Most VOC measurements were lower than 65.67 ppb during the data collection (Figure 6c). The descriptive statistics of VOC are as follows: average, 40.97 ppb; maximum, 160.67 ppb; minimum, 0 ppb; and standard deviation, 34.81 ppb. Federally enforceable guideline values have not been clearly defined for ambient VOCs because multiple VOCs are simultaneously present in the air, and outdoor VOC levels tend to be lower than those in occupational settings [32].

The data for PM_1_, PM_2.5_, and VOC were visualized on 2-D and 3-D maps based on their geographic coordinates and timesteps using GIS (Figure 6). PM_10_ maps were not included in this paper because PM_10_ concentrations were only slightly different from PM_2.5_ concentrations in these field data. It may be because the PM_10_ outputs are estimated from PM_0.5_, PM_1_, and PM_2.5_ measurements due to the limitations of today’s laser-based PM sensing technologies [29]. In addition, outdoor emission may be dominated by secondary organic aerosol from mobile emissions [33], and no coarse dust particles may have been detected.

The maps show that air pollution concentrations fluctuated over space and time along the volunteer’s GPS track. PM_1_ and PM_2.5_ data were classified using the same data classification scheme to facilitate the comparison (Figure 6a,b). PM_1_ and PM_2.5_ measurements showed similar spatial and temporal patterns overall. In addition to the 2-D maps, we presented the individual’s air quality data in a 3-D cube in which the horizontal surface represents space and the vertical axis represents time. The spatial range of the movement represents the geographical extent to which the individual traveled. The vertical axis represents the temporal progression of the travel. For further studies, this individual-level, fine spatiotemporal-scale data can be aggregated and combined with health data or other environmental, social, and behavioral variables using GIS. Additionally, although the maps presented in this paper are static, they can be easily converted to interactive web maps using web GIS technologies. The static or interactive web maps can be shared with research participants for visual communication. The visualization results illustrate that major roads with high traffic (three roads in the upper area on the maps) tend to have higher particle concentrations than smaller roads (two roads at the bottom), while VOC concentrations do not follow the same patterns.

The volunteer was only requested to charge and turn on the GeoAir device to collect and view air quality data because GeoAir does not require pairing with other devices or smartphone applications. A key recent trend of the Internet of Things (IoT) that comprises smart appliances is to replace the plastic buttons and LCD screens on standalone devices with smartphone applications. Therefore, many IoT devices, including low-cost air pollution sensors, utilize applications to pair the devices with mobile phones. While such emerging technologies have brought numerous opportunities for scientific research, they may not always work as intended when used in citizen science projects that involve elderly citizens or people who are less comfortable with new technologies or do not own smartphones of sufficient quality. This raises questions about the broader applicability of such devices beyond scientific needs and about their potential as tools to engage citizens while ensuring volunteer diversity and inclusivity, as well as socially and ethically responsible knowledge production [22]. GeoAir, an all-in-one device, is a highly promising tool because it can be used by all citizens regardless of their technical experience. The scope of a citizen science project using GeoAir can range from simple participatory data collection to data visualization/analysis using the data generated from the device.

GeoAir has several limitations worth noting. First, the performance of GeoAir, as would that of a typical low-cost sensor, may be influenced by weather conditions [34,35]. Due to the relatively limited robustness compared to fixed-site national air quality monitoring stations, it may be less suitable for regulatory purposes for which high reliability and accuracy are critical [36]. Further studies are needed to evaluate the effects of relative humidity and temperature through field tests in a wide range of weather conditions. Second, GeoAir does not provide a comprehensive air monitoring solution because sensors for other pollutants, such as carbon monoxide, ozone, or nitrogen dioxide, are not included in the device. However, integrating multiple air sensors is not the major consideration in this study because it may significantly increase the weight and size of the device and users’ burden. Therefore, we prioritized the pollutants that are most important to human health and that the general public is most concerned about in their everyday life. Third, the current firmware of the device does not support differential data recording intervals by activity, location, and time. Future studies may consider developing algorithms to automatically detect human activities (e.g., sleeping, sitting, walking, biking, or driving) or microenvironments (e.g., indoors, outdoors) and differentiate the recording intervals accordingly to save battery more effectively. Finally, although GeoAir is portable and wearable, it may be less suitable for long-term everyday use due to its weight (238 g) and size (14 × 5.3 × 3.5 cm), when compared to other commercial devices for which the weight and size were the most critical design consideration. However, because it is only slightly bigger and heavier than a typical smartphone, it may not significantly increase the burden for users.

## 4. Conclusions

This study proposes a fully integrated air-sensing device, GeoAir, to address the limitations of off-the-shelf air-sensing devices currently on the market. This novel instrument is fundamentally different from existing consumer devices because it was primarily designed for geospatial assessments of personal exposure and community-engaged research. The integrated position tracking system allows researchers to collect georeferenced air quality data at an individual level without a separate GPS tracking device and to visualize the personalized data on maps. These visual representations of data are effective tools for communicating with citizens about local air quality, their daily exposure, and potential health impacts.

The reliability of the data that GeoAir generates enables researchers to employ it for scientific research. The PM sensor used in GeoAir demonstrates strong performance when compared to a reference instrument. The VOC sensor shows reasonable accuracy, but future studies that test it with other types of VOC are needed to further assess the sensor performance. The credible, high-resolution, georeferenced air pollution data generated from the devices can form the basis of various individual- or community-level studies, as well as mitigation and intervention strategies. Such data could be utilized to identify potential sources of pollution, understand the impact of exposure to air pollution on respiratory diseases (e.g., asthma), develop strategies to educate the public and reduce air pollution and health risks, and evaluate the effectiveness of the strategies [37].

Due to its ease-of-use, GeoAir also holds significant potential for community-based participatory research or public participation GIS (PPGIS) projects that focus on environmental justice or health disparities. Its user friendliness substantially reduces the burden of participation and facilitates the engagement of underserved communities, particularly people who are technically less capable [11]. Engaging members of these communities in monitoring the environmental conditions of their immediate surroundings or communities can empower them to modify their behaviors to reduce air pollution and exposure in their daily lives. The knowledge gained may also encourage them to advocate for changes to eliminate polluting sources that are disproportionally distributed to their communities [38]. These efforts would contribute to improving community health and providing positive health outcomes for all individuals.

## Figures and Tables

**Table 1:** Slope, intercept and R^2^ for PM_1_, PM_2.5_, and PM_10_.

Particle Size	Slope	Intercept (μg/m^3^)	R^2^
PM_1_	0.77	−0.01	0.99
PM_2.5_	0.82	−0.11	0.99
PM_10_	0.95	−0.46	0.98

## Data Availability

The datasets generated from the current study are available from the corresponding author on reasonable request.
